# Facilities in Molecular Biomarkers in Cardiology

**DOI:** 10.3390/biom14081025

**Published:** 2024-08-17

**Authors:** Pietro Scicchitano, Matteo Cameli

**Affiliations:** 1 Cardiology Section, Hospital “F. Perinei” ASL BA, 70022 Altamura, Italy; 2 Department of Medical Biotechnologies, Division of Cardiology, University of Siena, 53100 Siena, Italy; matteo.cameli@unisi.it

This Special Issue of *Biomolecules*, entitled “Molecular Biomarkers in Cardiology 2022–2023”, presents a comprehensive collection of research and reviews exploring the rapidly evolving field of cardiovascular biomarkers.

The identification of the perfect biomarkers in cardiovascular diseases is a challenging issue [1]. The application of advances in technologies and artificial intelligence will surely provide new insights in this field [2]. Nevertheless, new research is needed in order to validate all the newly proposed early detection methods and treatments for cardiovascular diseases.

This Editorial highlights some key contributions that underscore the significance of molecular biomarkers in diagnosing, prognosticating, and understanding cardiovascular diseases (CVDs).

Zhou et al. [3] employed advanced metabolomics to identify potential biomarkers for the early diagnosis of ST-segment elevation myocardial infarction (STEMI) and non-ST-segment elevation myocardial infarction (NSTEMI). By analyzing the differential metabolites (DMs) in patients, the researchers provide insights into specific metabolic changes that can serve as early indicators of acute myocardial infarction (AMI), which remains a leading cause of mortality globally. This work emphasizes the potential of metabolomics in enhancing diagnostic precision and timeliness in AMI cases. Vargas-Alarcón et al. [4] investigated the genetic underpinnings of atherosclerosis. They focused on FOXA3 polymorphisms and their association with metabolic parameters and subclinical atherosclerosis, highlighting the genetic contributions to cardiovascular risk and the importance of personalized medicine approaches.

This is interesting in relation to new advances in lipid disorders connected to atherosclerotic diseases. Di Pietro et al. [5] delved into the role of sphingolipids in cardiovascular diseases. The authors discussed how these bioactive lipids contribute to CVD pathogenesis and their potential as biomarkers, offering a fresh perspective on cardiovascular risk assessment and therapeutic targets.

In parallel to ACS, lipids might also demonstrate a certain role in heart failure. Degoricija et al. [6] investigated the prognostic value of very-low-density lipoprotein (VLDL) cholesterol levels in acute heart failure (AHF). Their findings suggest that the VLDL cholesterol content can predict the one-year mortality in AHF patients, underscoring the role of lipid metabolism in heart failure prognosis. This study provides a compelling argument for integrating lipid biomarkers into clinical practice for improved management and risk stratification of heart failure patients.

Cardiovascular risk factors are not only related to lipids. Hypertension is one of the leading causes of death worldwide and the early identification of the damage it causes is the mainstay for clinicians [7]. Kumric et al. [8] explored the association between serum catestatin levels and primary hypertension (PH). Catestatin, a neuroendocrine peptide, is shown to correlate with blood pressure and arterial stiffness, indicating its potential as a biomarker for PH. This research adds a valuable piece to the puzzle of hypertension pathophysiology and opens new avenues for diagnostic and therapeutic strategies.

Indeed, an obscure issue in cardiovascular diseases is pulmonary arterial hypertension (PAH). Correale et al. [9] provided an update on circulating biomarkers related to PAH. They discussed the biomarkers involved in vasoconstriction, vascular remodeling, and inflammation, offering insights into the multifaceted nature of PAH and the potential for novel diagnostic and therapeutic targets.

Heart failure is a worrisome condition when dealing with biomarkers [10,11]. Licordari et al. [12] discussed the limitations of traditional biomarkers like natriuretic peptides and explore emerging alternatives. This comprehensive review emphasizes the need for novel biomarkers that can provide more detailed insights into heart failure’s pathophysiology, enabling more effective management and treatment.

Finally, the intersection of cardiology and oncology is examined by Attanasio et al. [13]. This review highlights the importance of biomarkers in detecting and managing cardiotoxicity induced by cancer therapies, a growing concern in the treatment of cancer patients. The authors underscore the need for integrated approaches to monitor and mitigate cardiovascular risks in these patients.

This Special Issue, “Molecular Biomarkers in Cardiology 2022–2023”, presents cutting-edge research that underscores the critical role of molecular biomarkers in advancing cardiovascular medicine. From metabolomics and genetic studies to emerging biomarkers in heart failure and cardio-oncology, these contributions highlight the diverse and dynamic landscape of cardiovascular biomarker research. By improving diagnostic accuracy, prognostic assessments, and personalized treatment strategies, these studies pave the way for better cardiovascular health outcomes.

## References

[1] Sahagun D., Zahid M. (2023). Cardiac-Targeting Peptide: From Discovery to Applications. Biomolecules.

[2] Urban S., Błaziak M., Jura M., Iwanek G., Ponikowska B., Horudko J., Siennicka A., Berka P., Biegus J., Ponikowski P. (2022). Machine Learning Approach to Understand Worsening Renal Function in Acute Heart Failure. Biomolecules.

[3] Zhou J., Hou H.T., Song Y., Zhou X.L., Chen H.X., Zhang L.L., Xue H.M., Yang Q., He G.W. (2024). Metabolomics Analysis Identifies Differential Metabolites as Biomarkers for Acute Myocardial Infarction. Biomolecules.

[4] Vargas-Alarcón G., Fragoso J.M., Ramírez-Bello J., Posadas-Sánchez R. (2022). FOXA3 Polymorphisms Are Associated with Metabolic Parameters in Individuals with Subclinical Atherosclerosis and Healthy Controls-The GEA Mexican Study. Biomolecules.

[5] Di Pietro P., Izzo C., Abate A.C., Iesu P., Rusciano M.R., Venturini E., Visco V., Sommella E., Ciccarelli M., Carrizzo A. (2023). The Dark Side of Sphingolipids: Searching for Potential Cardiovascular Biomarkers. Biomolecules.

[6] Degoricija V., Klobučar I., Potočnjak I., Dokoza Terešak S., Vidović L., Pregartner G., Berghold A., Habisch H., Madl T., Frank S. (2022). Cholesterol Content of Very-Low-Density Lipoproteins Is Associated with 1-Year Mortality in Acute Heart Failure Patients. Biomolecules.

[7] Cameli M., Lembo M., Sciaccaluga C., Bandera F., Ciccone M.M., D’Andrea A., D’Ascenzi F., Esposito R., Evola V., Liga R. (2020). Identification of cardiac organ damage in arterial hypertension: Insights by echocardiography for a comprehensive assessment. J. Hypertens..

[8] Kumric M., Vrdoljak J., Dujic G., Supe-Domic D., Ticinovic Kurir T., Dujic Z., Bozic J. (2022). Serum Catestatin Levels Correlate with Ambulatory Blood Pressure and Indices of Arterial Stiffness in Patients with Primary Hypertension. Biomolecules.

[9] Correale M., Tricarico L., Bevere E.M.L., Chirivì F., Croella F., Severino P., Mercurio V., Magrì D., Dini F., Licordari R. (2024). Circulating Biomarkers in Pulmonary Arterial Hypertension: An Update. Biomolecules.

[10] Scicchitano P., Massari F. (2023). The role of bioelectrical phase angle in patients with heart failure. Rev. Endocr. Metab. Disord..

[11] Massari F., Scicchitano P., Iacoviello M., Passantino A., Guida P., Sanasi M., Piscopo A., Romito R., Valle R., Caldarola P. (2020). Multiparametric approach to congestion for predicting long-term survival in heart failure. J. Cardiol..

[12] Licordari R., Correale M., Bonanno S., Beltrami M., Ciccarelli M., Micari A., Palazzuoli A., Dattilo G. (2024). Beyond Natriuretic Peptides: Unveiling the Power of Emerging Biomarkers in Heart Failure. Biomolecules.

[13] Attanasio U., Di Sarro E., Tricarico L., Di Lisi D., Armentaro G., Miceli S., Fioretti F., Deidda M., Correale M., Novo G. (2024). Cardiovascular Biomarkers in Cardio-Oncology: Antineoplastic Drug Cardiotoxicity and Beyond. Biomolecules.

